# Red nodule on the scrotum

**DOI:** 10.1016/j.jdcr.2025.02.038

**Published:** 2025-03-20

**Authors:** Kana Osawa, Akane Minagawa, Masao Fukuzawa, Ryuhei Okuyama

**Affiliations:** a Department of Dermatology, Shinshu University School of Medicine, Matsumoto, Japan; b Division of Dermatology, Ina Central Hospital, Ina, Japan

**Keywords:** hairpin vessel, poroma, verruciform xanthoma, yellow globules

## Clinical presentation

**Fig 1:**
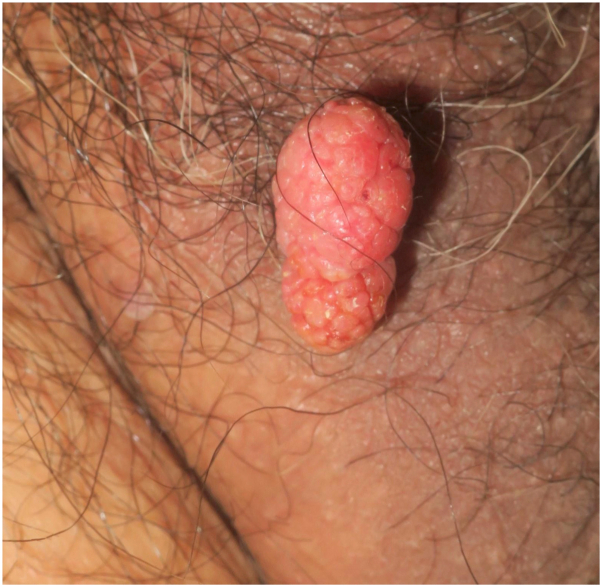
Clinical image of verruciform xanthoma on the right scrotum.

A 72-year-old man sought treatment for a red nodule on the scrotum that had gradually enlarged with pain over the course of 2 years. The nodule was pedunculated in shape, 10 × 20 × 15 mm in size, with a papillomatous surface (Fig 1).

## Dermatoscopic appearance

**Fig 2:**
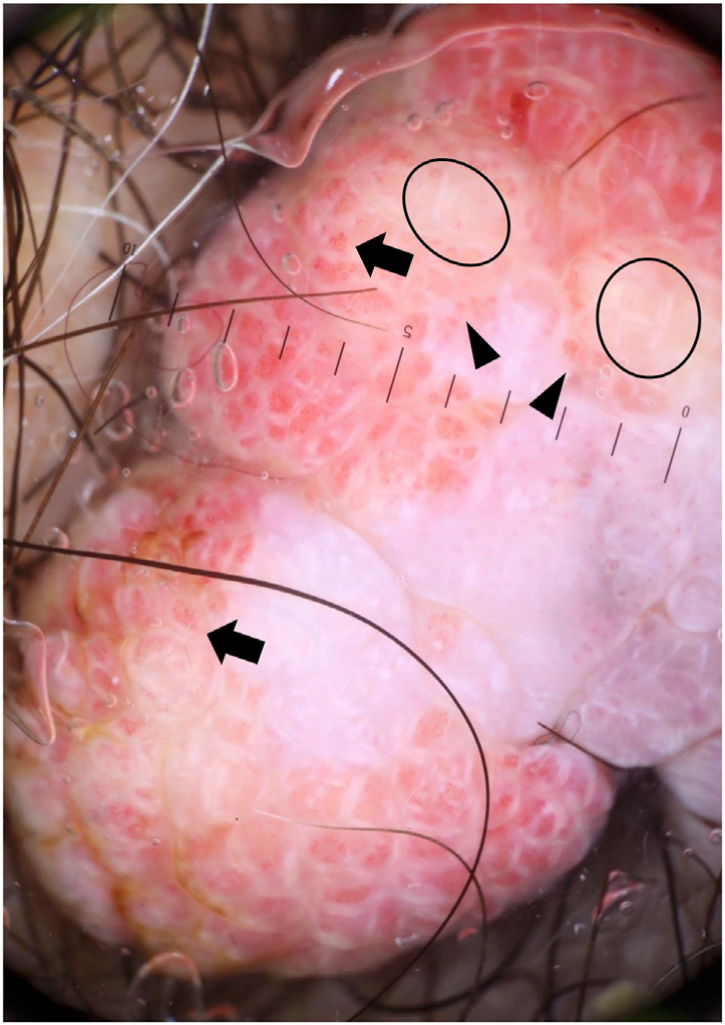
Dermatoscopic image of verruciform xanthoma on the right scrotum. Circles indicate yellow dots. Arrowheads and arrows indicate hairpin vessels and flower-like vessels, respectively (original magnification: ×10).

Dermatoscopic examination revealed combination of white networks and red globules on most areas of the nodule. Elongated hairpin vessels and flower-like vessels were visible within the red globules. Yellow dots and debris were scattered over the lesion (Fig 2).

## Histologic diagnosis

**Fig 3:**
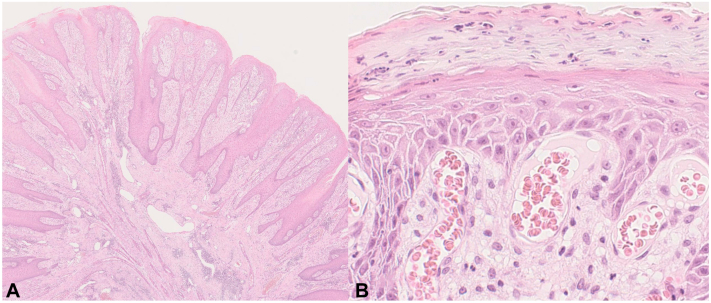
Histopathologic features of verruciform xanthoma. (**A**) The epidermis was irregularly thickened with papillomatous change. (**B**) Numerous foam cells and dilated capillaries were observed in the papillary dermis. (**A** and **B** hematoxylin-eosin stain; original magnification: **A**, ×20; **B,** ×400).

The lesion was diagnosed as verruciform xanthoma (VX) (Fig 3).

**Key message**

VX clinically presents as a light yellow or reddish nodule and is most commonly found in the anogenital area among middle-aged and elderly individuals. The differential diagnoses of VX include verruca vulgaris, poroma, and xanthomas. Histologically, VX is characterized by papillary proliferation of the epidermis and numerous foam cells in the dermal papilla. Capillary dilations are occasionally detected in the papillary dermis as well.^1^ The dermatoscopic findings of white networks and red globules with elongated hairpin and flower-like vessels correspond histopathologically to the papillary proliferation of the epidermis and capillary dilation in the dermis, respectively, which are shared with verruca vulgaris and poroma. However, yellow dots are absent in those diseases since they are related to the foam cells in VX.^2^ Meanwhile, xanthomas dermatoscopically present with yellow dots but rarely exhibit white networks, red globules, elongated hairpin vessels, or flower-like vessels.^1^ The characteristic combination of dermatoscopic findings in VX may help clinicians with diagnostic accuracy.

## Conflicts of interest

None disclosed.
